# Structure-function analysis of human stomatin: A mutation study

**DOI:** 10.1371/journal.pone.0178646

**Published:** 2017-06-02

**Authors:** Stefanie Rungaldier, Ellen Umlauf, Mario Mairhofer, Ulrich Salzer, Christoph Thiele, Rainer Prohaska

**Affiliations:** 1 Department of Medical Biochemistry, Max F. Perutz Laboratories, Medical University of Vienna, Vienna, Austria; 2 Center for Physiology and Pharmacology, Medical University of Vienna, Vienna, Austria; 3 LIMES Institute, Rheinische Friedrich-Wilhelms-University Bonn, Bonn, Germany; University of British Columbia, CANADA

## Abstract

Stomatin is an ancient, widely expressed, oligomeric, monotopic membrane protein that is associated with cholesterol-rich membranes/lipid rafts. It is part of the SPFH superfamily including stomatin-like proteins, prohibitins, flotillin/reggie proteins, bacterial HflK/C proteins and erlins. Biochemical features such as palmitoylation, oligomerization, and hydrophobic “hairpin” structure show similarity to caveolins and other integral scaffolding proteins. Recent structure analyses of the conserved PHB/SPFH domain revealed amino acid residues and subdomains that appear essential for the structure and function of stomatin. To test the significance of these residues and domains, we exchanged or deleted them, expressed respective GFP-tagged mutants, and studied their subcellular localization, molecular dynamics and biochemical properties. We show that stomatin is a cholesterol binding protein and that at least two domains are important for the association with cholesterol-rich membranes. The conserved, prominent coiled-coil domain is necessary for oligomerization, while association with cholesterol-rich membranes is also involved in oligomer formation. FRAP analyses indicate that the C-terminus is the dominant entity for lateral mobility and binding site for the cortical actin cytoskeleton.

## Introduction

Stomatin is a 31 kDa monotopic integral membrane protein that is palmitoylated, forms homo-oligomers, and associates with cholesterol-rich membrane domains, also known as lipid rafts [1]. It was first identified in the “band 7” region of human erythrocyte membrane proteins [2–5]. Due to the lack of this protein in red cells of Overhydrated Hereditary Stomatocytosis (OHSt) patients, it was termed “stomatin” [4]. However, the stomatin knockout mouse was viable and did not show stomatocytosis [6]. The lack of this protein in OHSt erythrocytes appears to be due to mistrafficking during terminal erythropoiesis [7]. Human stomatin is ubiquitously expressed in all tissues; highly in hematopoietic cells, relatively low in brain [8,9]. It is associated with the plasma membrane and cytoplasmic vesicles of fibroblasts, epithelial and endothelial cells [1], notably late endosomes [10], lipid droplets [11], and specialized endosomes/granules in hematopoietic cells [12,13]. In resting blood platelets, stomatin is mainly associated with α-granules and relocalizes to the plasma membrane upon activation [12]. Similarly, in neutrophils, stomatin is associated with azurophil granules, but also other specific granules [13], and is likewise relocated to the plasma membrane upon activation [1]. Stomatin is also expressed in placental cells, where it may play an important role in trophoblast differentiation [14], and in bone, where it promotes osteoclastogenesis [15]. Trafficking of stomatin to the plasma membrane appears to follow the Golgi-pathway [16], while endocytosis most probably follows a clathrin-independent endocytosis pathway similar to caveolin-1 [17] and flotillins [18]. When stomatin and stomatin-like protein 1 (SLP-1) are co-expressed, they form a complex at the plasma membrane that is targeted to late endosomes due to a Tyr-dependent targeting signal on SLP-1 and appears to be involved in cholesterol transfer and transport [19].

In the human genome, five related genes are coding for stomatin (*STOM*), stomatin-like proteins 1–3 (*STOML1*, *STOML2*, *STOML3*), and podocin (*NPHS2*) [1,20]. Homologues of stomatin are found in archaea, bacteria, and all higher eukaryotes, thus revealing an ancient protein family [21,22]. Moreover, a superfamily based on the presence of the conserved PHB/SPFH domain includes stomatins, prohibitins, flotillins, and HflK/C proteins [23–25], and more recently the erlins [26]. Stomatin and stomatin-like proteins interact with various ion channels in cholesterol-rich membrane domains [27–37] and regulate channel activities in a cholesterol-dependent manner [33]. In particular, human stomatin interacts with the glucose transporter GLUT1, thereby regulating glucose and dehydroascorbate influx into erythrocytes [38]. These findings suggest a function for stomatin as an integral scaffolding protein.

Structural features of human stomatin are the N-terminal, 24-residue, basic domain that is phosphorylated at Ser-10 by a cAMP-dependent protein kinase [5], followed by a 29-residue hydrophobic domain that is associated with the cytoplasmic face of the membrane and palmitoylated at Cys-30 [39]. This hydrophobic intramembrane domain (residues 26–54) contains the conserved residue Pro-47, which is essential for the rare monotopic membrane protein structure of stomatin and stomatin-like protein 3 (SLP-3) [40] and other stomatin-like proteins [33,41]. A similar proline residue, responsible for the monotopic structure, is also present in caveolin-1 [42,43]. Adjacent to the intramembrane domain, apparently located at the apolar-polar interphase, a short sequence, KIIKEYERAII (residues 55–65), shows similarity to the cholesterol-binding CRAC (cholesterol recognition/interaction amino acid consensus) motif (L/V)-X_1−5_-(Y)-X_1−5_-(K/R) [44,45] and reverse CRAC (“CARC”) motif (K/R)-X_1−5_-(Y/F)-X_1−5_-(L/V) [46]. Moreover, there is also an alternative CARC motif, RAIIFRL (residues 62–68) overlapping with the CRAC motif. The following, highly conserved PHB/SPFH domain (residues 57–256) [23] is palmitoylated at Cys-87, which is essential for correct membrane attachment [16,39]. The PHB/SPFH core domain has been elucidated by NMR and crystallography showing a compact ellipsoid structure composed of α-helices on one side and β-sheet on the other [47–49]. Downstream of this core domain, there is a well-conserved, prominent, 44-residue long, Ala-Glu-rich rod-like α-helix (residues 201–244), flanked by residues Pro-200 and Pro-245, that interacts with an identical helix to form an anti-parallel coiled-coil structure [47]. Interestingly, Pro-245 gives rise to a kink in the extending helical structure. The C-terminal end contains the oligomerization and lipid raft-association (ORA) domain (residues 265–273), which is essential for homo-oligomer formation and association with cholesterol-rich membranes [10,50,51]; it also contains the overlapping CARC motif KNSTIVFPL (residues 263–271). The motif IDML (residues 273–276) was predicted to bind to a PDZ3 protein [22], while the adjacent region (residues 273–287) was shown to interact with the LanC-like protein LANCL1 [52,53].

Comparing the crystal structures of the PHB/SPFH core domains from *Pyrococcus horikoshii* and mouse stomatin [47,48], some differences are showing that are based on varying interacting domains. While the archaeal PHB/SPFH domains form trimers with head-to-tail orientation and interact via coiled-coils [47], the mouse domains interact with their C-termini to form banana-shaped dimers similar to BAR domains and the N-termini of adjacent dimers are bound to each other by hydrophobic interactions yielding linear oligomers [48]. Whichever structure may turn out to be physiologically relevant, both structures have revealed crucial amino acid residues, which imply functional roles.

In this study, we have generated point mutants by exchanging some of these crucial residues in human stomatin and created deletion mutants. We have expressed these in the human cell line A431, which is low in endogenous stomatin, and analyzed the subcellular localization, stability, dynamics, and biochemical properties such as oligomerization, cholesterol binding, and association with lipid raft-like detergent-resistant membranes (DRMs). We identified stomatin as a cholesterol binding protein and noticed that both the CRAC/CARC (residues 55–68) and ORA/CARC (residues 263–273) domains are equally important for the association with cholesterol-rich domains. This association with cholesterol-rich membranes also appears to be a condition for oligomer formation, while the coiled-coil domain is clearly essential for oligomerization. At the plasma membrane, lateral mobility measurements revealed the interaction of the C-terminus (residues 264–288) with the actin cytoskeleton. Our data are in accordance with the previously proposed function of stomatin as an integral scaffolding protein.

## Materials and methods

### Materials

Research chemicals were obtained from Sigma. Cell lines A431 and COS-7 were purchased from ATCC. [^3^H]photocholesterol was synthesized as described by Thiele et al. [54]. Monoclonal mouse antibody against c-myc was obtained from the Developmental Studies Hybridoma Bank (DSHB) and rabbit anti-GFP was from Abcam. The mouse monoclonal antibody against human stomatin (GARP-50) was described [2].

### Cell culture

Human squamous epithelial carcinoma cells (A431) and the derived stably transfected cell lines were maintained in Dulbecco’s modified Eagle’s medium (DMEM) with high glucose, supplemented with 10% fetal bovine serum (Sigma) and antibiotics under standard conditions.

### Cholesterol depletion or loading

For cholesterol depletion or loading studies, cells were incubated with methyl-β-cyclodextrin (MβCD) or the cholesterol-MβCD complex in Hank’s balanced salt solution (HBSS) supplemented with 25 mM HEPES, pH 7.4. Briefly, the cell medium was removed, cells were washed twice with Tris-buffered saline (TBS) and incubated with cholesterol depletion buffer (0.5% MβCD in HBSS, 25mM HEPES, pH 7.4) or loading buffer (1 mM cholesterol in 0.5% MβCD in HBSS, 25mM HEPES, pH 7.4) at 37° for 30 min. Cells were washed once with TBS and HBSS and were imaged in HBSS, 25 mM HEPES, pH 7.4 on a Zeiss 510 META confocal laser scanning microscope (CLSM).

### DNA mutagenesis and cell transfection

To express human wild-type (WT) stomatin, with or without myc-tag, the eukaryotic expression vector pEF-Puro.PL3 was used as described previously [10,50]. The cDNA coding for stomatin was generated by PCR and directionally cloned into the unique restriction sites SpeI/EcoRV. To generate GFP-tagged WT or mutant stomatin, the cDNAs for WT or truncated stomatin extended by an Ala-linker (Gly-Ala-Ala-Ala) at the C-terminus were generated by PCR using the following primers: ST1Kozak.EcoRI, 5’-TACGGAATTCCGCCACCATGGCCGAGAAGCGGCACACAC-3’; ST287Ala.BamHI, 5’-TGCGGGATCCGGCGGCAGCGGCTCCGCCTAGATGGCTGTGTTTTGCC-3’; ST21Kozak.EcoRI, 5’-TACGGAATTCCGCCACCATGAGCCCCAGTAAGGGCCTTGGAC-3’; and ST262Ala.BamHI, 5’-TGCGGGATCCGGCGGCAGCGGCTCCTTTCTCAGCAGCAATGGTGGTC-3’. cDNAs were directionally cloned into the unique restriction sites EcoRI/BamHI of pEGFP-N3. Deletions and amino acid exchanges were generated by PCR, using mutagenic oligonucleotides containing the desired mutations (S1 Table) as primers for PCR reactions as described previously [11,19,51]. All constructs were verified by sequencing (LGC Genomics). A431 cells were stably or transiently transfected using Metafectene^™^ (Biontex) or calcium phosphate (Promega). After selection in medium containing 2 μg/ml puromycin or 700 μg/ml G418, individual stable clones were picked by trypsinization using cloning rings. Expression of the recombinant protein was screened by Western blot analysis using anti-GFP and anti-stomatin antibodies. GFP-expressing clones were further screened by fluorescence microscopy.

### Confocal laser scanning microscopy (CLSM)

Confocal fluorescence microscopy of paraformaldehyde-fixed specimens was essentially performed by standard methods as described previously [10,11]. Pictures were acquired using the Zeiss LSM 510 META CLSM and the corresponding software.

### Fluorescence Recovery After Photobleaching (FRAP) analysis

A431 cell lines stably transfected with GFP-tagged WT or mutated stomatin were chosen that showed plasma membrane expression of the construct. The cells were seeded on 35 mm glass-bottom dishes (MatTek Corp., Ashland, MA) and cultivated for 24 h. For cholesterol depletion or loading, cells were incubated with methyl-β-cyclodextrin (MβCD) or the cholesterol-MβCD complex in Hank’s balanced salt solution (HBSS). For disruption of actin microfilaments, cells were treated with 10 μM cytochalasin D (cytoD) in medium for 30 min. FRAP analysis was performed on the Zeiss LSM 510 META confocal microscope equipped with a stage heater at 37°, as described previously [51]. Analyses were repeated at least 20-times for each cell line. Fluorescence intensity data of whole cell ROI, bleached ROI, and background ROI were quantified using the Zeiss software package. Recovery curves were bleach- and background-corrected and the mobile fractions and recovery halftimes were calculated as described [55]. Recovery data, derived from ≥20 experiments, were fit to the equation y = y_o_ + a(1-e^-kt^) with GraphPad Prism scientific graphing software to calculate the rate constant k. The recovery halftime was calculated as t_1/2_ = ln2/k.

### Density gradient centrifugation

Cell membranes were prepared, solubilized, and subjected to density gradient ultracentrifugation as described [50]. Briefly, the membranes of confluent A431 clones (one 15-cm dish each) stably expressing GFP-tagged WT or mutant stomatin were solubilized in 200μl of 1% Triton X-100 in TNE buffer (20 mM Tris-Cl, pH 8.0, 130 mM NaCl, 5 mM EDTA) containing protease inhibitors (10 μg/ml aprotinin and leupeptin, 1 μg/ml pepstatin A, and 1 mM phenylmethylsulfonyl fluoride) at room temperature for 10 min. The solubilized proteins were applied on top of a linear density gradient (15–50% sucrose) and centrifuged in an SW40 rotor (Beckman) at 40,000 rpm and 4°C for 19 h. Nineteen fractions were collected from the top and subjected to SDS-PAGE and Western blotting. The sucrose gradient was regularly checked by refractometry. Marker proteins were run in parallel.

Flotation analysis was carried out as described [10]. Briefly, cell membranes of confluent A431 clones (one 15-cm dish each) were prepared by homogenization and centrifugation. These were solubilized in 280 μl of ice-cold TNE buffer containing 1% Triton X-100 for 10 min. The lysate was adjusted to 50% sucrose by the addition of 490 μl of 80% sucrose in TNE and overlaid with 3 ml of 35% sucrose followed by 1.2 ml of 5% sucrose in the same buffer. After ultracentrifugation at 48,000 rpm and 4°C in an SW 55 rotor (Beckman) for 18 h, 9 fractions were collected from the top and analyzed by Western blotting. Detergent-resistant membrane (DRM) components were identified in the low-density fractions 2 and 3, whereas Triton X-100-soluble proteins were found in the dense fractions 5 to 9.

### Binding of photocholesterol

COS-7 cells were transiently transfected with WT and mutated stomatin constructs, respectively, with or without myc-tags, and subjected to photocholesterol cross-linking according to the method of Thiele et al. [54,56]. Briefly, the cells were incubated in 10 cm dishes with 10 μCi [^3^H]photocholesterol in DMEM at 37°C for 15 min and irradiated for 1 min with a 500 W beam of filtered UV-light (λ > 310 nm) of a high-pressure mercury lamp at 4°C to crosslink photocholesterol to cholesterol-binding proteins. The cells were lysed, stomatin was immunoprecipitated with anti-stomatin antibody GARP-50 and analyzed by SDS-PAGE and autoradiography. Protein expression levels were estimated by immunoblotting with GARP-50 and densitometry of stomatin bands.

### Western blot analysis

Fractions collected from the sucrose gradients were analyzed by 10% SDS-PAGE and blotted onto nitrocellulose by standard methods as described previously [50]. HighPrecisionProtein^™^ Standards (Bio-Rad) were used as molecular weight markers. After blocking, the blots were incubated with anti-stomatin antibody GARP-50, anti-GFP, or anti-myc for 1 h. After washing, the blots were incubated with anti-mouse or anti-rabbit horseradish peroxidase conjugate and the SuperSignal^™^ chemiluminescent substrate (Pierce).

### Statistical analysis

Densitometric values of Western blot or autoradiographic bands were obtained by using ImageJ software and analyzed statistically with the IBM SPSS program. Mean values, standard deviation, and standard error were calculated and p-values were determined by two-tailed t-test.

## Results

### Mutation and expression of GFP-tagged stomatin

**Fig 1:**
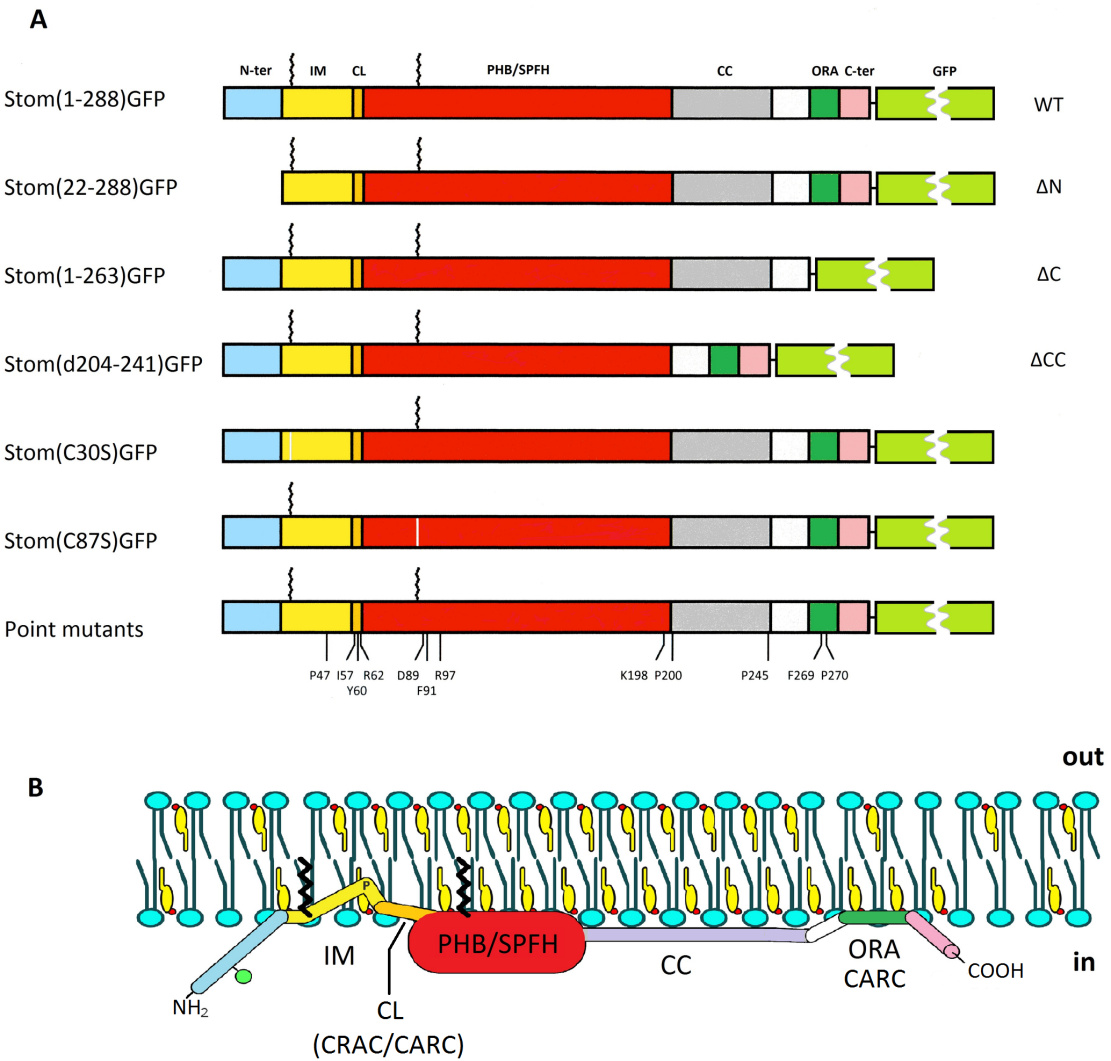
Schematic structure of GFP-tagged wildtype and mutant stomatin. (**A**) Schematic model of wildtype (WT) stomatin, composed of the N-terminal region (N-ter), intramembrane domain (IM), cholesterol recognition/interaction amino acid consensus (CRAC)-like motif (CL), prohibitin homology domain (PHB), also known as stomatin, prohibitin, flotillin, HflK/C (SPFH) domain, coiled-coil domain (CC), oligomerization and lipid raft-association domain (ORA), and C-terminal domain (C-ter). Palmitate residues bound to Cys-30 and Cys-87 are symbolized by zigzag lines. Stomatin mutants are shown that are deleted at the N-terminus (ΔN), C-terminus (ΔC), and coiled-coil domain (ΔCC), respectively. The positions of exchanged amino acid residues in point mutants are marked. Exchange of Cys-30 or Cys-87 for Ser abolished palmitate bonding. (**B**) Hypothetical model of a monomeric wildtype stomatin in association with a biological membrane. Sidedness is marked by “in” (cytoplasmic) and “out” (extracellular or luminal). The color code denotes the domains as illustrated in (**A**). The green ball at the N-terminal region symbolizes the phosphorylation site at Ser-10; the “P” at the kink within the hydrophobic IM domain marks residue Pro-47, which is responsible for the monotopic membrane protein structure. The model is roughly drawn according to known and estimated sizes; the N-terminal region is α-helical (E. Umlauf, unpublished results), the PHB/SPFH core domain is 5 nm in length and 2 nm in height, while the coiled-coil domain is 6 nm long [47]. CARC denotes a reversed CRAC motif; there are three CARC motifs, two overlapping with the CRAC-like (CL) and one overlapping with the ORA motif. Schematic models of the most remarkable mutants are shown in S4 Fig.

To study the relevance of some prominent structural features of stomatin, we deleted parts of the molecule and exchanged single amino acids. The recent elucidation of the 3-dimensional structure of the highly conserved PHB/SPFH core of archaeal and mouse stomatin [47,48] highlighted several amino acid residues that implicated a structural and functional role. Moreover, the finding that the conserved, long α-helix adjacent to the PHB/SPFH domain was involved in coiled-coil interactions [47] gave a hint as to the formation of stomatin oligomers. Therefore, we exchanged the residues in question and deleted the long α-helix to study their effects on the structure and function of stomatin. An overview of the deletions and amino acid exchanges that we have introduced into GFP-tagged stomatin is given in Fig 1.

#### Generation and stable transfection of GFP-tagged stomatin mutants

All of the constructs shown in Fig 1 were stably transfected in the human carcinoma cell line A431 that is known for low expression of endogenous stomatin [51]. Single cell-derived clones were isolated, expanded and analyzed for stable expression of the GFP fusion proteins. To get a variety of expression levels, we pooled various isolated clones to populations.

#### Subcellular localization of expressed GFP-tagged stomatin mutants

In a variety of cell lines, stomatin is localized to the plasma membrane (PM) and the late endosomal/lysosomal compartment [10,11,19,57]. Therefore, we started out by comparing our stable mutant cell lines with respect to these compartments.

The localization in A431 cells of WT stomatin, Stom(1–288)GFP, and the deletion mutants Stom(22–288)GFP (ΔN), Stom(1–263)GFP (ΔC), and Stom(d204-241)GFP (ΔCC), depicted in S1 Fig, appeared as previously shown for endogenous or myc-tagged stomatin in various cell lines [10,57], namely at the PM and cytoplasmic vesicles. However, several mutants were unable to target PM, namely Pro47Ser, Cys87Ser, Asp89Ala, Arg97Ala, Lys198Ala, and Pro270Ala. Other mutants, such as Ile57Ala, Tyr60Ala, Pro200Ala, and Pro245Ala showed reduced PM staining (S1 Fig). The complete localization data of the stomatin mutants are summarized in S2 Table.

### Cholesterol binding of wildtype and mutant stomatin

**Fig 2:**
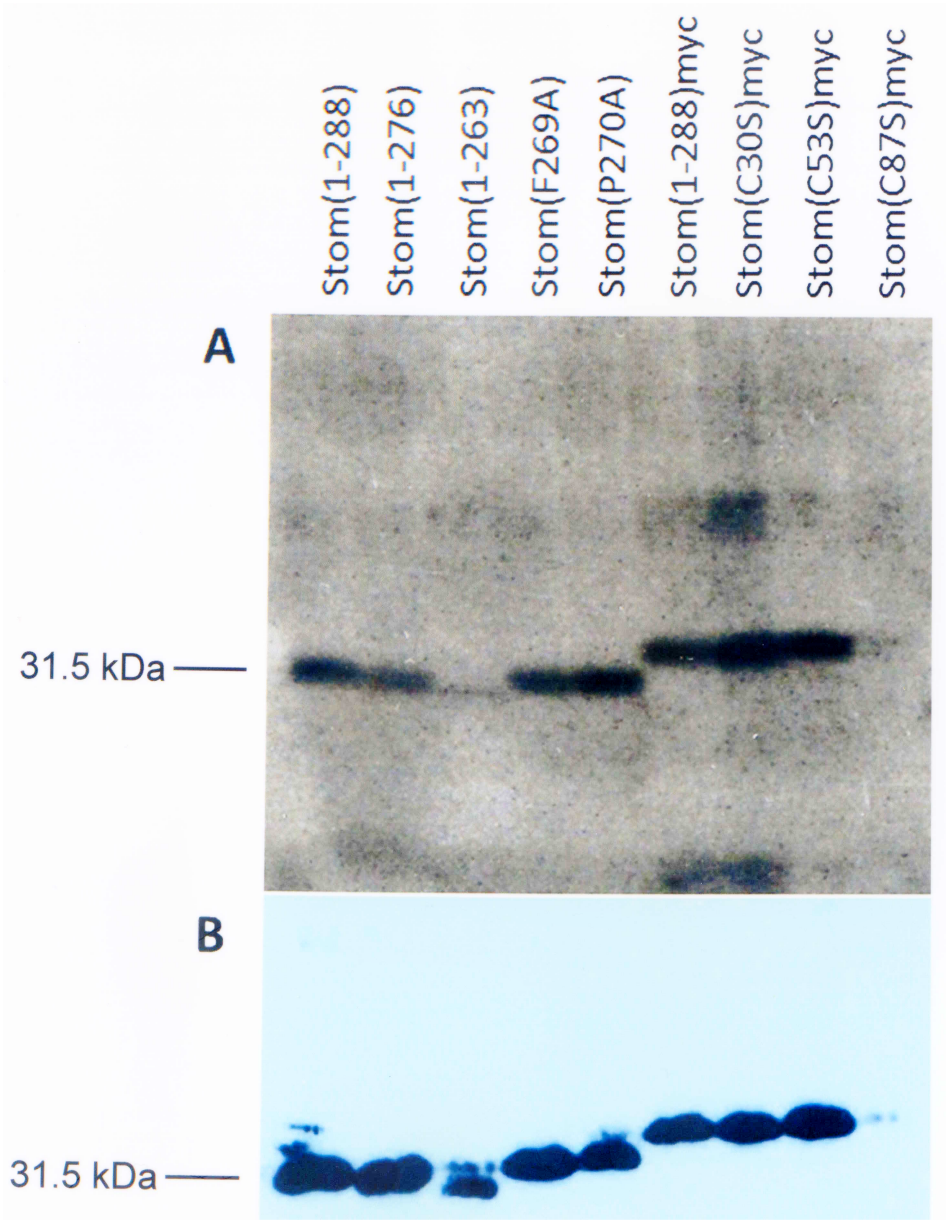
Binding of [^3^H]photocholesterol to wildtype and mutant stomatin. COS-7 cells were transiently transfected with WT or mutant stomatin constructs. Subsequently they were incubated with a photoactivatable, radioactive cholesterol derivative ([^3^H]photocholesterol) and irradiated with UV light to crosslink [^3^H]photocholesterol to respective binding proteins. The cells were solubilized and stomatin was immunoprecipitated by monoclonal anti-stomatin antibody GARP-50. (**A**) SDS-PAGE and autoradiography revealed cholesterol-binding to WT and mutant stomatin. (**B**) The expression level of the constructs was determined by immunoblotting with monoclonal anti-stomatin antibody GARP-50.

Wildtype and mutant stomatin constructs with or without myc-tag were transiently expressed in COS-7 cells, incubated with [^3^H]-labeled photocholesterol and irradiated with UV light for crosslinking. The expressed proteins were immunoprecipitated with anti-stomatin antibody and the covalently bound [^3^H]photocholesterol was identified by autoradiography of SDS-PAGE gels. To estimate the expression levels of WT and mutant stomatin, immunoblotting with anti-stomatin antibody was performed. Radioactive bands were observed for WT stomatin and essentially all mutants (Fig 2).

Quantitative data were assessed by densitometry of autoradiography and Western blot bands (Table 1) and by calculating the ratios of these data. The expression levels of mutants varied largely, thereby changing the ratio of bound cholesterol to each stomatin mutant. The C-terminal deletion mutant Stom(1–263) bound only one third of WT-bound cholesterol (Table 1) suggesting the loss of a binding site at the C-terminal end (residues 264–288). Interestingly, the values of Stom(F269A) and Stom(P270) were quite different; while cholesterol-binding to F269A was slightly reduced, binding to P270A was enhanced almost twofold, when compared to WT. The myc-tagged constructs showed higher cholesterol-binding, particularly the Cys-mutants; however, the results of mutants Stom(C53S)myc and Stom(C87S)myc were not significant due to exceedingly varying ratios.

**Table 1:** Densitometric analysis of [^3^H]photocholesterol-binding to wildtype and mutant stomatin.

Construct	Cholesterol-binding (%)		Expression (%)		Ratio (cholesterol/protein)		p-value
	Mean	SD	Mean	SD	Mean	SD	
**Stom(1–288) (WT)**	100	─	100	─	1.00	─	─
**Stom(1–276)**	70	7	118	12	0.60	0.09	0.160
**Stom(1–263)**	12	2	42	8	0.30	0.09	0.005
**Stom(F269A)**	80	2	110	15	0.73	0.11	0.055
**Stom(P270A)**	98	31	51	6	1.90	0.39	0.058
**Stom(1–288)myc**	125	20	63	4	2.00	0.40	0.048
**Stom(C30S)myc**	175	60	60	15	2.92	0.62	0.033
**Stom(C53S)myc**	161	27	65	16	2.63	1.14	0.130
**Stom(C87S)myc**	26	21	17	15	6.79	10.14	0.427

Densitometric values obtained from autoradiography and Western Blot bands were normalized to WT stomatin by setting the absorbance of the WT band to 100%. Mean values and standard deviation (SD) were calculated from the values of 3 independent experiments (n = 3). Cholesterol-binding relative to expression level was calculated by forming the ratio. The p-values indicate the significance of the differences between values of mutants and WT.

### Oligomerization and DRM-association of GFP-tagged wildtype and mutant stomatin

Hallmarks of stomatin structure and function are the oligomeric nature [50,51] and association with lipid raft-like detergent-resistant membranes (DRMs) [10,51]. Therefore, we analyzed the GFP-tagged stomatin mutants to evaluate their ability to form oligomers and associate with DRMs.

#### Oligomerization of GFP-tagged wildtype and mutant stomatin

**Fig 3:**
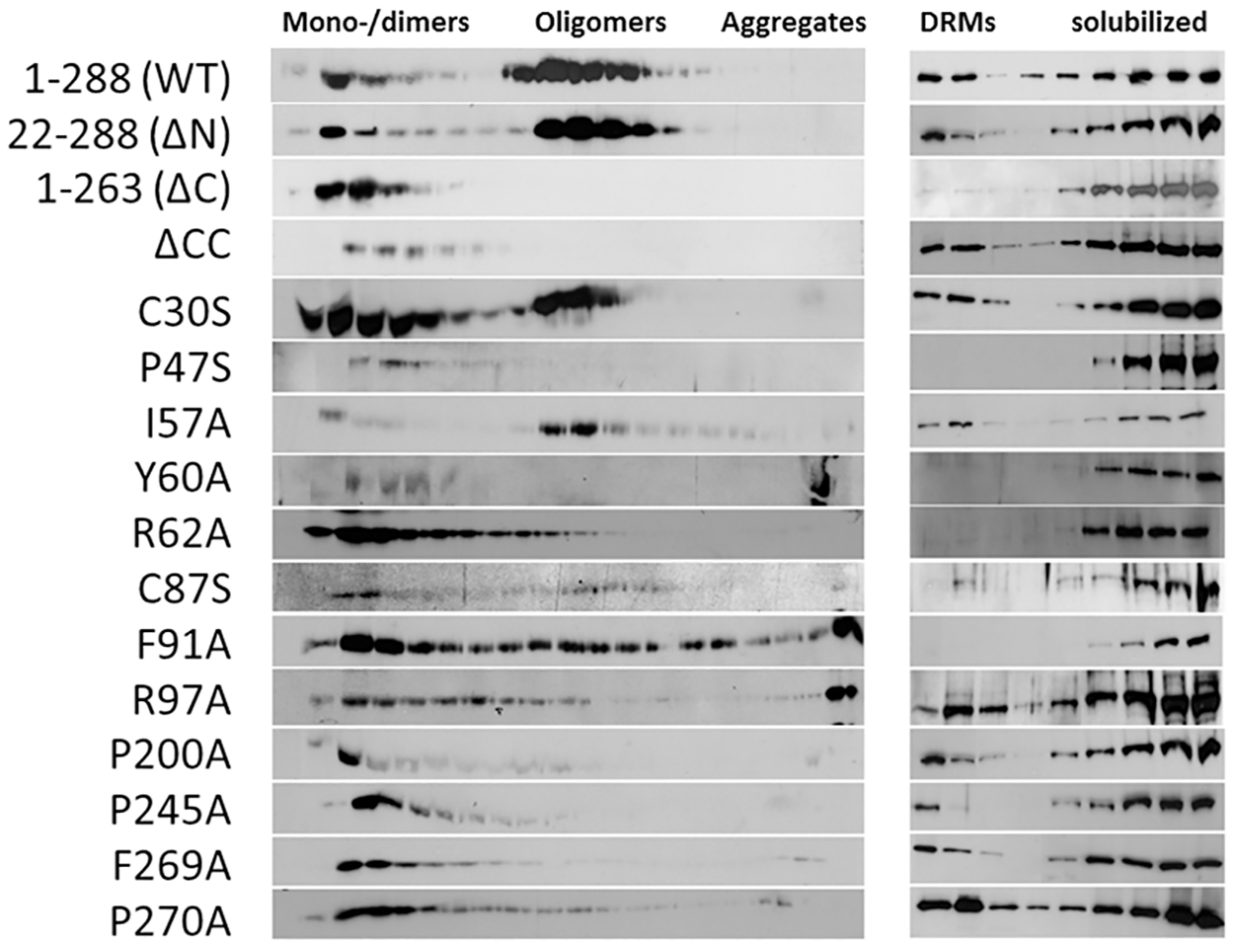
Oligomerization and DRM-association of GFP-tagged wildtype and mutant stomatin. A431 cells stably expressing GFP-tagged WT or mutant stomatin were solubilized and subjected to linear density gradient centrifugation to estimate molecular size (left panel) or step density gradient centrifugation to determine DRM-association (right panel). Gradient fractions were analyzed by SDS-PAGE and proteins were identified by immunoblotting with anti-GFP. The linear 15–50% sucrose gradient was verified by refractometry and calibrated by marker proteins. SDS-PAGE was performed by running molecular weight markers in parallel. GFP-tagged stomatin constructs showed values of about 70 kDa except for the Pro47Ser mutant, which was estimated at 80 kDa, as predicted due to glycosylation [40].

We analyzed oligomers by linear density gradient ultracentrifugation (Fig 3, left panel, S2 Fig, and Table 2). Due to the large size of the oligomers, we used higher than usual concentrations, i.e. 15–50% sucrose gradient. WT stomatin yielded oligomers in the range of 500–700 kDa but also a varying amount of mono- or dimers (70–150 kDa) estimated at 10–50% of total stomatin (n = 5). It has to be taken into account that the large GFP-tag may at least partially affect stomatin oligomerization or oligomer stability in solution; however, a few percent of monomer are also found in endogenous stomatin and untagged oligomeric mutants [50,51]. The N- and C-terminal deletion mutants, ΔN and ΔC, showed similar distributions as the previously described myc-tagged mutants [50], that is oligomerization like WT and inability to oligomerize, respectively (Fig 3, left panel, S2 Fig). The coiled-coil deletion mutant, ΔCC, yielded only mono-/dimers. Cys30Ser showed roughly equal parts being mono-/dimeric and oligomeric, while the other Cys mutant, Cys87Ser, appeared similar but was largely degraded, as noted before [39]. Pro47Ser was mono-/dimeric (Fig 3, left panel, S2 Fig), as anticipated [40]. The CRAC/CARC mutants Tyr60Ala and Arg62Ala yielded mainly mono-/dimers (Fig 3, left panel). Tyr60Ala appeared prone to aggregation, as seen by precipitated material at the bottom of the gradient (Fig 3, left panel, S2 Fig). Exchange of Ile-57 did not affect oligomerization (Fig 3, left panel); the Ile57Ala mutant may have been rescued by Ile-56. Phe91Ala showed a mixed phenotype, partly being mono-/dimeric, partly oligomeric (Fig 3, left panel); however, it also appeared prone to aggregation, because of precipitated material in the bottom fraction of the gradient (Fig 3, left panel, S2 Fig). The continuous distribution range from monomers to high oligomers suggests that continuous aggregation of Phe91Ala was taking place during the 19 h centrifugation time. Exchange of Arg-97 led to impaired oligomerization (Fig 3, left panel) and aggregation (Fig 3, left panel, S2 Fig). Substitution of proline residues, Pro-200 and Pro-245, which are flanking the coiled-coil domain, and Pro-270 of the ORA domain, yielded mono-/dimeric proteins (Fig 3, left panel, S2 Fig). The ORA/CARC mutant Phe269Ala was also unable to oligomerize, as described [51]. Densitometry of Western blot bands obtained from gradient fractions showed the distribution of mono-/dimers (fractions 1–6), oligomers (fractions 7–18), and aggregates (fractions 19). Relative amounts (% of total) are listed and visualized (Table 2, S2 Fig).

**Table 2:** Molecular size distribution of GFP-tagged wildtype and mutant stomatin.

Constructs	Monomers (%)		Oligomers (%)		Aggregates (%)		p-values (oligomers)
	Mean	SD	Mean	SD	Mean	SD	
**1–288 (WT)**	28.4	6.5	71.4	6.6	0.2	0.1	─
**22–288 (ΔN)**	21.0	3.4	78.9	3.4	0.1	0.1	0.072
**1–263 (ΔC)**	98.3	2.1	1.5	1.8	0.2	0.4	<0.001
**ΔCC**	92.4	3.9	7.3	4.2	0.3	0.5	<0.001
**Cys30Ser**	53.6	5.6	45.9	5.1	0.5	0.5	0.001
**Pro47Ser**	89.6	3.7	10.2	3.5	0.2	0.3	<0.001
**Ile57Ala**	21.2	5.6	77.3	5.0	1.5	1.1	0.172
**Tyr60Ala**	50.5	12.4	10.4	7.5	39.1	9.1	<0.001
**Arg62Ala**	74.2	6.0	25.6	5.8	0.2	0.2	<0.001
**Cys87Ser**	46.7	13.1	48.8	10.4	4.5	2.2	0.005
**Phe91Ala**	42.4	7.4	42.7	9.3	14.9	1.9	0.001
**Arg97Ala**	54.5	11.9	18.7	14.6	26.8	2.8	<0.001
**Pro200Ala**	81.5	7.2	15.3	6.9	3.2	2.2	<0.001
**Pro245Ala**	85.0	9.4	14.0	8.1	1.0	1.3	<0.001
**Phe269Ala**	88.4	1.4	8.3	1.9	3.3	0.8	<0.001
**Pro270Ala**	75.8	0.9	20.5	1.3	3.7	0.9	<0.001

WT, wildtype; ΔN, N-terminal deletion; ΔC, C-terminal deletion; ΔCC, coiled-coil deletion. The relative amounts of mono-/dimers, oligomers, and aggregates (in % of total) were determined by densitometry of respective Western blot bands (Fig 3, left panel). Data were obtained from ≥3 experiments (n ≥ 3). Mean values, standard deviations (SD), and p-values are given. The p-values indicate the significance of the differences between oligomer values of mutants and WT.

#### DRM-association of GFP-tagged wildtype and mutant stomatin

We compared the stomatin mutants with respect to DRM-association using sucrose density step gradient ultracentrifugation (Fig 3, right panel). WT stomatin showed a distribution similar to endogenous stomatin [10,51], partly floating to the top but partly also being dissolved in the detergent Triton X-100 (Fig 3, right panel, and S3 Fig; Table 3). While the N-terminal truncation mutant (ΔN) behaved like WT, the C-terminal truncation (ΔC) led to absence from the floating DRM-fraction, as previously shown for the untagged or myc-tagged proteins [10,51]. Deletion of the coiled-coil domain (ΔCC) did not interfere with DRM-association. Apparently, there is no need for stomatin to oligomerize in order to associate with cholesterol-rich membranes. Palmitoylation is thought to be necessary for DRM-association; however, both Cys30Ser and Cys87Ser partially floated to the top (Fig 3, right panel, and S3 Fig). Exchange of Pro-47 led to exclusion from DRMs (Fig 3, right panel, and S3 Fig), as previously reported for this substitution [40,41]. The mutants with exchanged residues Tyr-60 and Arg-62 of the CRAC/CARC motif were not able to associate with DRMs; however, exchange of Ile-57 had a slight, enhancing effect on DRM-association (S3 Fig), possibly due to rescue by Ile-56. Mutant Phe91Ala was not floating with DRMs (Fig 3, right panel, and S3 Fig; Table 3), which might indicate misfolding of the molecule and precipitation, as noticed in the linear gradient (Fig 3, left panel, and S2 Fig). Exchange of the structurally important proline residues Pro-200, Pro-245, and Pro-270, only affected DRM-association of Pro245Ala (Fig 3, right panel, and S3 Fig), whereas oligomerization was impaired for all of them (Fig 3, left panel, and S2 Fig; Table 2). The mono-/dimeric ORA/CARC mutant Phe269Ala partially floated with DRMs in contrast to the untagged mutant reported previously [51]. The DRM-association data of all mutants are summarized in Table 3. Densitometry of Western blot bands obtained from gradient fractions showed the distribution of the DRM-associated WT or mutant stomatin (fractions 1–3), versus Triton X-100-soluble, non-DRM stomatin mutants (fractions 4–9). Relative amounts (% of total) are listed and visualized (Table 3, S3 Fig).

**Table 3:** Distribution of GFP-tagged wildtype and mutant stomatin between DRMs and non-DRMs.

Constructs	DRMs (%)		Non-DRMs (%)		p-values
	Mean	SD	Mean	SD	
**1–288 (WT)**	25.3	0.4	74.7	0.4	─
**22–288 (ΔN)**	25.0	3.1	75.0	3.1	0.841
**1–263 (ΔC)**	2.4	1.2	97.6	1.2	<0.001
**ΔCC**	21.3	1.6	78.7	1.6	0.003
**Cys30Ser**	19.9	1.1	80.1	1.1	<0.001
**Pro47Ser**	0.2	0.1	99.8	0.1	<0.001
**Ile57Ala**	35.6	0.6	64.4	0.6	<0.001
**Tyr60Ala**	5.0	4.3	95.0	4.3	<0.001
**Arg62Ala**	1.7	0.3	98.3	0.3	<0.001
**Cys87Ser**	13.2	6.1	86.8	6.1	0.009
**Phe91Ala**	1.1	1.0	98.9	1.0	<0.001
**Arg97Ala**	27.7	5.9	72.3	5.9	0.445
**Pro200Ala**	23.7	3.3	76.3	3.3	0.373
**Pro245Ala**	12.0	2.6	88.0	2.6	<0.001
**Phe269Ala**	19.4	3.0	80.6	3.0	0.008
**Pro270Ala**	33.5	3.1	66.5	3.1	0.002

WT, wildtype; ΔN, N-terminal deletion; ΔC, C-terminal deletion; ΔCC, coiled-coil deletion; DRMs, detergent-resistant membranes. The relative amounts (% of total) of WT or mutant stomatin in DRM-fractions (fractions 1–3) and non-DRM fractions (fractions 4–9) were determined by densitometry of respective Western blot bands (Fig 3, right panel). Data were obtained from ≥3 experiments (n ≥ 3). Mean values, standard deviations (SD), and p-values are given. The p-values indicate the significance of the differences between values of mutants and WT.

### Lateral mobility of GFP-tagged wildtype and mutant stomatin

**Fig 4:**
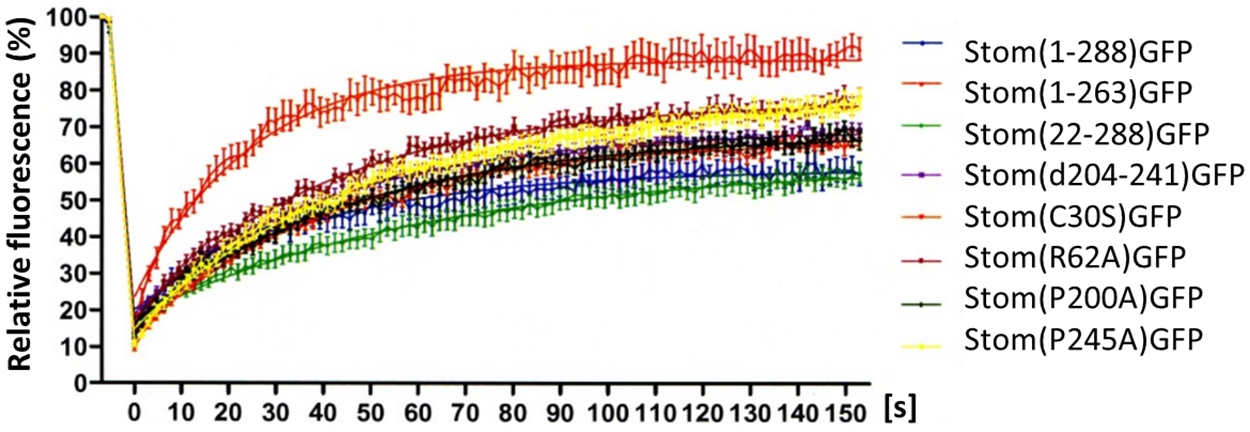
FRAP-analysis of GFP-tagged wildtype and mutant stomatin. A431 cells stably expressing GFP-tagged WT or mutant stomatin at the plasma membrane were analyzed by FRAP measurements. N ≥ 20. The data for mobile fractions and recovery halftimes are given in Table 4.

To interrelate the biochemical data with the situation in living cells, we analyzed the lateral mobility by Fluorescence Recovery After Photobleaching (FRAP) measurement of those mutants that were stably expressed at the PM (S1 Fig). Compared to WT, the C-terminal truncation mutant (ΔC) showed the largest mobile fraction (Fig 4, Table 4), in accordance with previous data [51]. ΔC neither formed oligomers nor associated with DRMs (Fig 3) and thus this lack of interaction may be the cause for the high lateral mobility in the membrane. Similarly, the second largest mobile fraction (MF) was observed for the CRAC-like mutant Arg62Ala that likewise did not oligomerize nor bind to DRMs. Comparing the data of all analyzed mutants, we found a reverse correlation between the size of MF and the ability to form oligomers or associate with DRMs (Table 4). The N-terminal deletion mutant (ΔN) and WT stomatin are both able to oligomerize and bind to DRMs [51] and they presented the lowest MFs, whereas the highest MFs were observed with mutants that lacked both oligomerization and DRM-association, i.e. Arg62Ala and ΔC (Fig 4, Table 4). It is easily conceivable that oligomerization and DRM-association constitute two kinds of membrane interactions that restrict lateral mobility.

**Table 4:** Lateral mobility of plasma membrane-bound GFP-tagged wildtype and mutant stomatin. Correlation of mobile fractions and recovery halftimes with the ability to oligomerize and/or associate with DRMs.

Stomatin mutant ^a^	Mobile fraction (MF)				Recovery halftime (t_1/2_)				Oligo	DRM
	Mean (%)	SE	ΔMF (%)	p-value	Mean [s]	SE	Δt_1/2_ [s]	p-value		
**ΔC**	88.6	5.1	+ 30.0	0.0001	17.4	5.1	- 9.3	0.1315	-	-
**Arg62Ala**	77.4	2.7	+ 18.8	0.0122	28.5	2.1	+ 1.8	0.2231	-	-
**Pro245Ala**	78.0	2.7	+ 19.4	0.0082	34.5	2.4	+ 7.8	0.6089	-	(+)
**ΔCC**	69.6	1.5	+ 11.0	0.1378	29.1	3.5	+ 2.4	0.4417	-	+
**Pro200Ala**	68.6	2.1	+ 10.0	0.2650	32.3	2.1	+ 5.6	0.2231	-	+
**Cys30Ser**	66.8	2.1	+ 8.2	0.4877	29.3	2.4	+ 2.6	0.2769	+	+
**ΔN**	60.3	2.4	+ 2.7	0.9560	44.5	8.3	+ 17.8	0.2056	+	+
**WT**	58.6	4.4	0.0	─	26.7	9.4	0.0	─	+	+

^a^ Mutants are listed roughly according to decreasing mobile fractions. MF, mobile fraction; t_1/2_, recovery halftime; ΔMF, MF-change of mutant versus WT; Δt_1/2_, t_1/2_-change of mutant versus WT; SE, standard error; ΔC, C-terminal deletion; ΔCC, coiled-coil deletion; ΔN, N-terminal deletion; WT, wildtype; Oligo, oligomerization; DRM, DRM-association; +, positive; (+), slightly positive (≈30% of WT); -, negative; ─, not applicable. Mean values, standard errors (SE), differences between mutants and WT values (ΔMF, Δt_1/2_), and p-values are given. Number of measurements for each value: n ≥ 20. The p-values indicate the significance of the differences between values of mutants and WT.

### Lateral mobility of GFP-tagged wildtype and mutant stomatin as a function of membrane cholesterol and actin cytoskeleton

Stomatin binds cholesterol (Fig 2), generates oligomers, associates with DRMs (Fig 3), and interacts with cortical actin microfilaments [57]. While the sites of oligomerization and/or DRM-association have been localized to the ORA region [51], coiled-coil domain, and CRAC/CARC motifs (Fig 3), the site of actin-binding is still unknown; however, a PDZ-protein binding motif near the C-terminal end [22] may be involved. To analyze the effects of the actin cytoskeleton and membrane cholesterol-levels on stomatin lateral mobility, we treated WT and mutant stomatin expressing cells with cytochalasin D (cytoD) and/or methyl-β-cyclodextrin (MβCD), with or without cholesterol.

**Fig 5:**
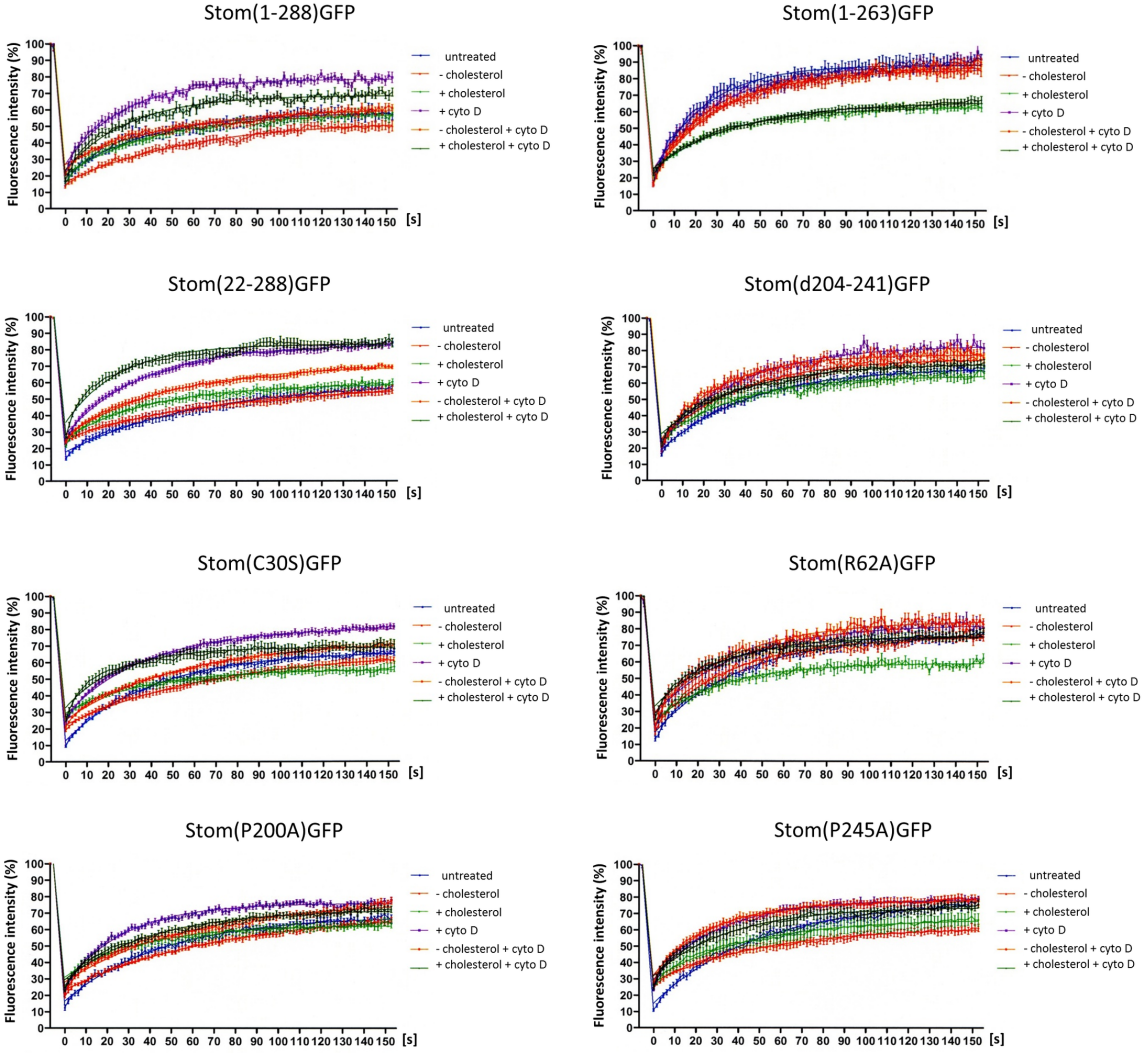
Lateral mobility of GFP-tagged wildtype and mutant stomatin. **Effects of cholesterol and cytochalasin D.** A431 cells stably expressing GFP-tagged WT or mutant stomatin at the plasma membrane were analyzed by FRAP measurements. The cells were either depleted of or loaded with cholesterol, treated with cytochalasin D (cytoD), or treated with a combination of both. N ≥ 20. The data for mobile fractions and recovery halftimes are given in Table 5.

In general, treatment of WT and mutants with cytoD showed increased MF and recovery (Fig 5, Table 5) suggesting that these proteins were dissociated from the cortical actin cytoskeleton or released from an actin-dependent PM compartment. According to the FRAP data, about 20% of WT stomatin were released from the immobile fraction by the treatment with cytoD and most likely were associated with the actin cytoskeleton. Similar values were found for ΔN, ΔCC, and Cys30Ser, while smaller differences were seen with Arg62Ala and Pro200Ala. There was no effect of cytoD on ΔC and Pro245Ala MFs, suggesting that these mutants were not associated with the actin cytoskeleton. Recovery halftimes showed a remarkable increase in the lateral diffusion of WT, ΔN, Pro200Ala, and Pro245Ala, while the other mutants presented marginal increases.

**Table 5:** Lateral mobility of plasma membrane-bound GFP-tagged wildtype and mutant stomatin. Effects of cholesterol and cytochalasin D.

Stomatin mutant	Treatment	Mobile fraction (MF)				Recovery halftime (t_1/2_)			
		Mean (%)	SE	ΔMF (%)	p-value	Mean [s]	SE	Δt_1/2_ [s]	p-value
**WT**	none	58.6	4.4	─	─	26.7	9.4	─	─
**WT**	-chol	53.9	7.7	- 4.7	0.9217	41.4	16.7	+14.7	0.1211
**WT**	+chol	57.9	2.7	- 0.7	0.3673	29.4	4.2	+ 2.7	0.3573
**WT**	+cytoD	79.0	1.7	+20.4	0.0022	18.8	2.5	- 7.9	0.0330
**WT**	-chol +cytoD	61.3	3.8	+ 2.7	0.8306	33.8	11.9	+ 7.1	0.7506
**WT**	+chol +cytoD	69.1	2.1	+10.5	0.1830	20.8	4.6	- 5.9	0.1137
**ΔN**	none	60.3	2.4	─	─	44.5	8.3	─	─
**ΔN**	-chol	58.2	3.1	- 2.1	0.8330	49.9	12.1	+ 5.4	0.3480
**ΔN**	+chol	59.3	3.1	- 1.0	0.6186	28.9	13.5	- 15.6	0.6259
**ΔN**	+cytoD	83.2	2.2	+22.9	<0.0001	25.6	1.4	- 18.9	0.0007
**ΔN**	-chol +cytoD	70.7	5.4	+10.4	0.0248	34.1	14.3	- 10.4	0.7731
**ΔN**	+chol +cytoD	83.3	2.3	+23.0	<0.0001	17.7	3.0	- 26.8	0.0003
**ΔC**	none	88.6	5.1	─	─	17.4	5.1	─	─
**ΔC**	-chol	85.7	2.8	- 2.9	0.1971	22.2	2.1	+ 4.8	0.7227
**ΔC**	+chol	63.0	2.5	- 25.6	<0.0001	23.9	7.0	+ 6.5	0.2423
**ΔC**	+cytoD	88.6	3.0	0.0	0.5478	21.8	5.1	+ 4.4	0.9478
**ΔC**	-chol +cytoD	87.1	2.0	- 1.5	0.2276	22.6	1.5	+ 5.2	0.7656
**ΔC**	+chol +cytoD	66.1	2.0	- 22.5	<0.0001	28.0	2.4	+10.6	0.3684
**ΔCC**	none	69.6	1.5	─	─	29.1	3.5	─	─
**ΔCC**	-chol	75.4	2.4	+ 5.8	0.0407	26.5	3.3	- 2.6	0.4965
**ΔCC**	+chol	64.9	2.6	- 4.7	0.1844	25.9	5.9	- 3.2	0.9956
**ΔCC**	+cytoD	83.5	3.4	+13.9	0.0004	26.3	4.1	- 2.8	0.5032
**ΔCC**	-chol +cytoD	77.7	2.3	+ 8.1	0.0120	19.9	2.6	- 9.2	0.0175
**ΔCC**	+chol +cytoD	72.2	2.0	+ 2.6	0.4856	26.5	2.6	- 2.6	0.2969
**Cys30Ser**	none	66.8	2.1	─	─	29.3	2.4	─	─
**Cys30Ser**	-chol	66.7	2.2	- 0.1	0.9245	49.0	4.6	+19.7	0.0003
**Cys30Ser**	+chol	55.8	3.7	- 11.0	0.0897	26.0	5.4	- 3.3	0.7465
**Cys30Ser**	+cytoD	81.1	1.9	+14.3	<0.0001	26.6	2.3	- 2.7	0.5719
**Cys30Ser**	-chol +cytoD	73.0	1.5	+ 6.2	0.0392	37.3	1.8	+ 8.0	0.0137
**Cys30Ser**	+chol +cytoD	69.2	2.5	+ 2.4	0.4429	17.5	2.6	- 11.8	0.0063
**Arg62Ala**	none	77.4	2.7	─	─	28.5	2.1	─	─
**Arg62Ala**	-chol	75.7	4.8	- 1.7	0.5329	25.7	2.8	- 2.8	0.3605
**Arg62Ala**	+chol	59.7	2.1	- 17.7	<0.0001	22.2	2.6	- 6.3	0.0254
**Arg62Ala**	+cytoD	83.2	4.4	+ 5.8	0.1802	26.8	4.2	- 1.7	0.5700
**Arg62Ala**	-chol +cytoD	85.4	2.7	+ 8.0	0.0289	26.1	3.3	- 2.4	0.5237
**Arg62Ala**	+chol +cytoD	75.8	2.4	- 1.6	0.7012	21.5	2.2	- 7.0	0.0750
**Pro200Ala**	none	68.6	2.1	─	─	32.3	2.1	─	─
**Pro200Ala**	-chol	70.7	5.7	+ 2.1	0.1618	50.6	3.7	+18.3	0.0002
**Pro200Ala**	+chol	62.8	3.0	- 5.8	0.3068	24.5	7.7	- 7.8	0.5346
**Pro200Ala**	+cytoD	76.3	1.2	+ 7.7	0.0089	19.3	1.3	- 13.0	0.0009
**Pro200Ala**	-chol +cytoD	77.6	2.6	+ 9.0	0.0048	40.7	5.2	+ 8.4	0.0055
**Pro200Ala**	+chol +cytoD	72.5	1.9	+ 3.9	0.2533	28.6	2.2	- 3.7	0.7598
**Pro245Ala**	none	78.0	2.7	─	─	34.5	2.4	─	─
**Pro245Ala**	-chol	61.8	2.1	- 16.2	0.0005	36.0	6.1	+ 1.5	0.3403
**Pro245Ala**	+chol	66.7	5.2	- 11.3	0.1293	30.9	3.5	- 3.6	0.0279
**Pro245Ala**	+cytoD	78.6	2.3	+ 0.6	0.9900	21.2	1.3	- 13.3	<0.0001
**Pro245Ala**	-chol +cytoD	77.7	2.6	- 0.3	0.9726	19.3	2.7	- 15.2	0.0005
**Pro245Ala**	+chol +cytoD	74.0	1.9	- 4.0	0.2923	24.8	3.4	- 9.7	0.0695

MF, mobile fraction; t_1/2_, recovery half-time; ΔMF, MF-change of treated versus untreated sample; Δt_1/2_, t_1/2_-change of treated versus untreated sample; SE, standard error; WT, wildtype; ΔN, N-terminal deletion; ΔC, C-terminal deletion; ΔCC, coiled-coil deletion; chol, cholesterol; cytoD, cytochalasin D. Mean values, standard errors (SE), differences between mutants and WT values (ΔMF, Δt_1/2_), and p-values are given. Number of measurements for each value: n ≥ 20. The p-values indicate the significance of the differences between values of treated versus untreated samples.

Cholesterol depletion of WT and mutants showed little changes in MF (Fig 5, Table 5). Only Pro245Ala presented a marked reduction in MF. In contrast, cholesterol depletion had some effect on the speed of recovery, particularly slowing down Cys30Ser and Pro200Ala. This effect is known and attributed either to the concomitant depletion of cholesterol and phosphatidylinositol(4,5)bisphosphate resulting in reorganization of the actin cytoskeleton [58–60] or formation of a solid-like phospholipid diffusion barrier [61]. The slower recovery after cholesterol depletion was partially reversed by concomitant cytoD treatment, indicating the involvement of cytoskeleton. For Cys30Ser, cholesterol depletion slowed down recovery (from 29.3 s to 49.0 s) but concomitant treatment with cytoD alleviated slowing to 37.3 s. Similarly, for Pro200Ala, the slowing of recovery due to cholesterol depletion (from 32.3 s to 50.6 s) was moderated (40.7 s) when cells were concomitantly treated with cytoD. However, rapid recovery was seen after cytoD treatment alone (19.3 s).

Cholesterol loading showed some reduction in MF, particularly of ΔC (-26%) and Arg62Ala (-17%). ΔC and Arg62Ala are mutants that do not associate with DRMs (Fig 3 and S3 Fig, Table 3), probably due to the loss of cholesterol binding sites, and neither oligomerize. Concomitant cytoD treatment of the mutants partially reversed the low MFs, suggesting that the actin cytoskeleton was involved, except for ΔC, which showed unchanged MF (Fig 5, Table 5), thus confirming that ΔC is not interacting with the actin cytoskeleton. Cholesterol loading had some impact on recovery halftimes, increasing lateral mobility, particularly of Arg62Ala and Pro245Ala. This effect of cholesterol loading had been attributed to endocytosis [60]; possibly, the increased speed reflects the cholesterol flow in the PM caused by increased endocytosis. Concomitant treatment with cytoD resulted in extremely rapid recovery of ΔN (17.7 s).

## Discussion

In this study, we used targeted mutation to evaluate the structural and functional significance of prominent domains and amino acid residues of the monotopic membrane protein stomatin. We selected these domains and residues on the basis of recent NMR and crystal structures of the PHB/SPFH domain [47–49] and our biochemical data [5,10,11,39,50,51]. Schematic models of WT and mutant stomatin showing the relevant domains and residues are depicted in Fig 1.

When we expressed the GFP-tagged mutants, we surveyed the subcellular localization and found that several mutants were unable to target the PM (S1 Fig, S2 Table). Other mutants, particularly Cys87Ser, were difficult to express at all. This may be due to gross structural changes and degradation or inefficient transport via the Golgi apparatus. Palmitoylation of Cys-87 has been shown to be essential for the attachment of the PHB/SPFH domain to the PM [16].

Stomatin-like proteins are known to bind cholesterol, which modifies interactions with ion channels thereby regulating channel activities [33,62]. To study whether stomatin itself binds cholesterol, we have used the photochemical crosslinking approach [54] comparing WT stomatin with various mutants (Fig 2, Table 1). We clearly showed covalent binding of [^3^H]photocholesterol to WT stomatin and all mutants (Fig 2). Comparing the relative amounts of bound cholesterol (Table 1), large differences were seen between different mutants. This may be explained by different affinities for cholesterol but also by large variations of mutant expression levels. The cholesterol-labeling data of all these mutants indicate that there is more than one cholesterol-binding site on stomatin. As pointed out, stomatin contains several domains or motifs that may function as cholesterol-binding sites [46], particularly in the CRAC and CARC regions. It is currently not clear, why myc-tagged stomatin shows higher cholesterol-binding than untagged stomatin (Fig 2, Table 1). Possibly, the modified C-terminus may cause a structural change that raises the affinity of cholesterol binding sites.

Apart from binding cholesterol, there are two other hallmarks of stomatin and similar monotopic proteins, such as flotillins and caveolins, namely the generation of oligomers and the association with lipid raft-like DRMs [10,50]. The basic mechanisms of the respective protein-protein and protein-lipid interactions are still unknown and probably need advanced methods for elucidation. Here, we used classical density gradient ultracentrifugation to estimate and compare the impacts of various mutations on oligomerization and DRM association (Fig 3, Tables 2 and 3, S2 and S3 Figs). Due to the relatively large GFP-tag, the changes seen were less pronounced than with untagged or myc-tagged proteins [51]; however, in principle, the data are in line with each other. One question arises, why in ΔC the lack of the ORA/CARC domain (residues 263–273) is causing the absence of this mutant from DRMs, when other putative cholesterol-binding sites such as the IM domain and adjacent CRAC/CARC-like region (residues 55–68) are still available. In this respect, is the C-terminal ORA/CARC domain more important for DRM-association than the upstream CRAC/CARC-like domain or is it necessary that these cholesterol-binding domains cooperate? Similarly, the exchange of the CRAC-like consensus residues Tyr-60 and Arg-62 led to absence of these mutants from DRMs despite the intact C-terminal ORA/CARC domain. This shows that both the upstream CRAC/CARC and downstream ORA/CARC domains are equally important and suggests that they do cooperate in cholesterol-rich membranes. Another question is, why should the lack of association with DRMs, as in the CRAC/CARC and ΔC mutants, lead to absence of oligomers? There may be a structural connection between the CRAC/CARC and ORA/CARC domains but it is currently unknown. In this context, several membrane proteins have been described to contain adjacent CRAC and CARC motifs, possibly for cholesterol-transfer [46]. Related to the above question is, how such a small domain as ORA/CARC (residues 263–273) may be causing two apparently unrelated effects, oligomerization and DRM association. Possibly, the major effect of the CRAC/CARC and ORA/CARC (ΔC) mutations is the disruption of stomatin binding to cholesterol-rich membranes/lipid rafts and this loss may in consequence prevent oligomerization due to unfavorable spatial alignment of monomers. Eventually, whole molecule structure analysis, e.g. cryoelectron microscopy, will give the answer to these questions.

Deletion of the coiled-coil domain (ΔCC) resulted in the absence of oligomers thereby confirming the interacting function of this domain [47], whereas DRM association was unaffected (Fig 3, Tables 2 and 3, S2 and S3 Figs), probably because the CRAC/CARC domains, particularly the possibly affected downstream ORA/CARC domain, remained intact (S4 Fig). Exchange of Pro-47 led to lack of oligomers and absence of DRM association as predicted for a single-pass transmembrane glycoprotein [40] (S4 Fig). Exchange of Phe-91, which may be involved in linear oligomerization of the PHB/SPFH domain, similar to mouse residue Leu-91 [48], led to an unstable molecule, with the tendency to aggregate; it did not associate with DRMs. Exchange of residues Pro-200 and Pro-245, which are flanking the coiled-coil domain, and Pro-270 of the ORA domain, prevented oligomerization, probably due to high flexibility of the coiled-coil domain and downstream region (S4 Fig), while DRM association was only affected in Pro245Ala. This suggests that in Pro200Ala and Pro270Ala, cholesterol-binding regions were left intact.

Comparing the three phenotypes of stomatin mutants, expression at the PM, oligomerization, and DRM association, showed that there is some intricate correlation between them (Table 6). Several mutants were unable to target the PM; however, those that were expressed at the PM neither required oligomerization nor association with DRMs. Some mutants, which did not oligomerize, were still able to associate with DRMs. However, all mutants that were oligomeric, also associated with DRMs. This suggests that DRM association precedes oligomerization or, in other words, DRM association is a necessary but not sufficient condition for stomatin oligomerization. It is conceivable that small, mono- or dimeric stomatin-cholesterol complexes may oligomerize to form large, stomatin-cholesterol/lipid aggregates. This is in line with the idea that small lipid rafts may coalesce to form large cholesterol-rich signaling platforms [63,64].

**Table 6:** Ability of GFP-tagged stomatin mutants to target the plasma membrane, form oligomers, and/or associate with DRMs.

Mutation	Affected domain	PM localization	Oligo-merization	DRM-association
**WT**	─	+	+	+
**ΔN**	N-terminal	+	+	+
**ΔC**	C-terminal	+	-	-
**ΔCC**	Coiled-coil	+	-	+
**Cys30Ser**	IM	+	+	+
**Pro47Ser**	IM	-	-	-
**Ile57Ala**	CRAC/CARC	(+)	+	+
**Tyr60Ala**	CRAC/CARC	(+)	-	-
**Arg62Ala**	CRAC/CARC	+	-	-
**Cys87Ser**	PHB/SPFH	-	(+)	+
**Phe91Ala**	PHB/SPFH	+	(+)*	-
**Arg97Ala**	PHB/SPFH	-	-	+
**Pro200Ala**	Coiled-coil	(+)	-	+
**Pro245Ala**	Coiled-coil	(+)	-	(+)
**Phe269Ala**	ORA/CARC	+	-	+
**Pro270Ala**	ORA/CARC	-	-	+

PM, plasma membrane; DRM, detergent-resistant membrane; WT, wildtype; ΔN, N-terminal deletion; ΔC, C-terminal deletion; ΔCC, coiled-coil deletion; IM, intramembrane domain; CRAC, cholesterol recognition/interaction amino acid consensus sequence; CARC, reverse CRAC motif; PHB, prohibitin homology domain; SPFH, stomatin-prohibitin-flotillin-HflK/C domain; ORA, oligomerization and lipid raft association domain; +, positive; -, negative; (+), lower than WT; (+)* denotes the unclear condition of Phe91Ala oligomers, which rather appear like unspecific aggregates (Fig 3, left panel; Table 2).

The in vitro biochemical characterization of oligomerization and DRM association does not directly reflect the situation of molecules in the living cell. Therefore, we complemented our biochemical data with Fluorescence Recovery After Photobleaching (FRAP) measurements to relate the lateral mobility of stomatin mutants to the phenotypes of varying oligomerization and/or DRM-association (Fig 4, Table 4). Of all analyzed mutants, the C-terminal deletion (ΔC) was outstanding with its high mobile fraction (MF) and rapid recovery. Because this mutant does not oligomerize nor associate with DRMs (Fig 3, Tables 2 and 3) [51], this lack of interaction with other monomers and/or cholesterol-rich membrane domains may cause this high lateral mobility. Comparing all FRAP data with the mutants’ biochemical characteristics, we saw that the highest MFs were measured for mutants that neither oligomerize nor associate with DRMs, while the lowest MFs were found for mutants that generate oligomers and bind to DRMs (Table 4). It is quite evident that such molecular interactions are reducing the lateral mobility; however, a major player in restricting lateral mobility is the actin cytoskeleton [65,66] and stomatin is known to interact with cortical actin microfilaments [57].

To evaluate the effects of the membrane actin meshwork on lateral mobility of stomatin mutants, we treated the cells with cytoD to disrupt the microfilaments. Moreover, to estimate the influence of cholesterol-rich lipid domains, we manipulated membrane cholesterol levels (Fig 5, Table 5). In general, treatment of cells with cytoD had the highest enhancing effect on MF of all mutants that had an intact C-terminus (Fig 5, Table 5), while it had no effect on mutants with lacking or impaired C-terminus (ΔC, Pro245Ala). This indicates that the C-terminal end (residues 264–288) contains an actin cytoskeleton binding site, as hypothesized [1]. Without an intact cortical cytoskeleton or a respective binding site, stomatin would be free to diffuse, as shown by the high MF and rapid recovery.

Depletion of cholesterol had little effect on MFs of mutants, except for the marked MF reduction of Pro245Ala and MF increase of ΔCC (Table 5); however, it resulted in slower recovery of WT and several mutants. This effect of cholesterol depletion appears to be a general phenomenon of all membrane proteins [60] and may be due to the reorganization of the actin cytoskeleton, because MβCD-treatment does not only sequester cholesterol but also phosphatidylinositol(4,5)bisphosphate [58,59], which is essential for actin filament organization. Alternatively, or additionally, a diffusion barrier of solid-like phospholipid domains may form in the absence of cholesterol [61] and thus restrict lateral mobility. Loading of cholesterol resulted in a marked increase in the immobile fraction, particularly of ΔC and Arg62Ala, two mutants that do not show oligomerization nor association with DRMs. In general, cholesterol loading shifts the subcellular distribution of membrane proteins and causes endocytosis [60]; possibly, the loss-of-function mutants ΔC and Arg62Ala are insensitive to membrane cholesterol flow and remain immobile, while mutants that bind cholesterol may follow the dynamic cholesterol gradient in the PM. In the case of Arg62Ala, the original MF was restored by cytoD, indicating that cytoskeleton was involved; however, the MF of ΔC was not restored by cytoD, according to the lack of actin-binding. Cholesterol loading may also lead to molecular crowding in the PM, increasing viscosity, and possibly immobilization of the overcrowded phase.

To sum up our data, we give here an overview of the induced structural changes and the resulting phenotypes (Table 7). We put the focus on six areas/domains of stomatin that appear functionally relevant, i.e. the N-terminus, IM-domain, CRAC/CARC motif, PHB/SPFH-domain, coiled-coil domain, and ORA/CARC domain. To illustrate the structural changes in stomatin, we show schematic models of the most remarkable mutant proteins (S4 Fig).

**Table 7:** Comparison of stomatin structural changes and functional consequences referring to wildtype.

Domain / Motif	Mutant	Structural change	Change of functional characteristics	Conclusion
**N-terminus**	ΔN	Loss of N-terminal domain	Highly similar to WT in all characteristics; slow diffusion, strongly enhanced by cytoD.	Oligomerization and DRM-association as described [51], see structural model (S4 Fig). Interaction with actin cytoskeleton, as WT.
**IM**	Cys30Ser	Loss of palmitoylation	Affected oligomerization; Reduced lateral speed after cholesterol-depletion.	Suggests a structural change impairing oligomerization. Cholesterol-depletion creates a barrier to Cys30Ala diffusion.
	Pro47Ser	Transmembrane glycoprotein	No PM-staining; no oligomer-formation; no DRM-association.	As to be expected from a normal transmembrane protein [40] (S4 Fig).
**CRAC/ CARC**	Ile57Ala	Loss of CRAC consensus residue	Weak PM-staining, enhanced oligomerization, enhanced DRM-association.	Suggests that Ile-56 rather than Ile-57 is the real CRAC consensus residue.
	Tyr60Ala	Loss of CRAC consensus residue	Weak PM-staining; affected oligomerization; aggregation/precipitation; no DRM-association.	Suggests a severe structural change. Loss of DRM-association is in line with a role of Tyr-60 in the CRAC consensus.
	Arg62Ala	Loss of CRAC consensus residue	No oligomerization; no DRM-association; large MF, strongly reduced by cholesterol-loading.	Loss of DRM-association is in line with a role of Arg-62 in the CRAC consensus. Large MF suggests low binding to cholesterol-rich membranes or cytoskeleton.
**PHB/ SPFH**	Cys87Ser	Loss of palmitoylation	Unstable, largely degraded; no PM-staining; affected oligomerization; affected DRM-association.	In line with the role of Cys-87-palmitate being the major anchor for the PHB/SPFH domain to the PM [16] (S4 Fig).
	Phe91Ala	Loss of putative head-to-head interaction site	Affected oligomerization; aggregation/precipitation; no DRM-association.	Suggests a role of Phe-91 in DRM-association. Tendency for aggregation suggests an unstable structure.
	Arg97Ala	Loss of prominent positive charge	No PM-staining; affected oligomerization.	In line with a role of Arg-97 as an interaction partner of (a) negatively charged PM component(s).
**Coiled-coil**	ΔCC	Loss of coiled-coil domain	No oligomer-formation; strongly enhanced MF after cytoD-treatment.	Lack of oligomerization verifies the proposed function in oligomerization via coiled-coil interaction (S4 Fig). Enhanced MF after cytoA suggests binding to cytoskeleton.
	Pro200Ala	Loss of structural fixpoint	Weak PM-staining; affected oligomerization; enhanced MF after cytoD-treatment.	Suggests a disability of the mutant to form oligomers via coiled-coil interaction due to higher flexibility (S4 Fig). Enhanced MF after cytoD suggests interaction with cytoskeleton.
	Pro245Ala	Loss of structural fixpoint	Weak PM-staining; affected oligomerization; reduced DRM-association; large MF, strongly reduced by cholesterol-loading; MF unchanged by cytoD.	Mutation effect suggests interference with ORA/CARC-binding to DRMs (S4 Fig). Pro-245 is an essential residue for downstream interactions with cortical actin cytoskeleton.
**ORA/ CARC**	ΔC	Loss of C-terminal domain	No oligomers; no DRM-association; largest MF, unchanged by cytoD; fastest diffusion; low cholesterol-binding (Table 1).	ORA characteristics as described [51] (S4 Fig). FRAP data indicate that the C-terminus is essential for actin-cytoskeleton-binding. Lack of DRM-association is in line with low cholesterol-binding.
	Phe269Ala	Loss of crucial aromatic residue	No oligomers; reduced DRM-association; reduced cholesterol-binding (Table 1).	ORA characteristics as described [51], except for partial DRM-association that suggests a tag-effect. Reduced DRM-association is in line with reduced cholesterol-binding.
	Pro270Ala	Loss of structural fixpoint	No PM-staining; affected oligomerization; enhanced DRM-association; enhanced cholesterol-binding (Table 1).	ORA characteristics as described [51]. Enhanced DRM-association is in line with stronger cholesterol-binding.

PM, plasma membrane; DRM, detergent-resistant membrane; ΔN, N-terminal deletion; ΔC, C-terminal deletion; ΔCC, coiled-coil deletion; IM, intramembrane domain; CRAC, cholesterol recognition/interaction amino acid consensus sequence; CARC, reverse CRAC motif; PHB, prohibitin homology domain; SPFH, stomatin-prohibitin-flotillin-HflK/C domain; ORA, oligomerization and lipid raft association domain; MF, mobile fraction.

In conclusion, we have shown that stomatin is a cholesterol binding protein and that one binding site is located within the C-terminal region (residues 264–288). Two regions of stomatin, the putative cholesterol binding sites, CRAC/CARC (residues 55–68) and ORA/CARC (residues 263–273), are equally responsible for the association with cholesterol-rich membranes. We conclude that these two regions are either structurally connected or cooperating. Moreover, this association with cholesterol-rich membranes appears to be a condition for oligomer formation possibly by creating a membrane microenvironment that induces oligomerization. For oligomerization, the coiled-coil domain is clearly essential, while the CRAC/CARC-residues Tyr-60, Arg-62, and the ORA/CARC domain, as well as Phe-91, may play an indirect role in oligomerization by their primary association with cholesterol-rich domains. The interaction of stomatin with the cortical actin cytoskeleton has been linked to the C-terminal region (residues 264–288).

## Supporting information

S1 Fig Subcellular localization of GFP-tagged wildtype and mutant stomatin. Confocal laser scanning microscopy (CLSM) of A431 cells stably expressing GFP-tagged wildtype (WT) stomatin shows the normal, dual localization to the plasma membrane (PM) and late endosomal/lysosomal compartment. Deletion of the N-terminus, C-terminus, or coiled-coil domain did not alter this localization and distribution. The point mutants Cys30Ser, Arg62Ala, Phe91Ala, and Phe269Ala, also showed the normal localization and distribution. Lack of PM staining or largely reduced staining was observed for Pro47Ser, Cys87Ser, Asp89Ala, Arg97Ala, Lys198Ala, and Pro270Ala, while weak staining of PM and preferential staining of cytoplasmic vesicles was visible in cells expressing Ile57Ala, Tyr60Ala, Pro200Ala, and Pro245Ala.
(TIF)

S2 Fig Distribution of monomers, oligomers, and aggregates of GFP-tagged WT and mutant stomatin. The relative amounts of mono-/dimers (fractions 1–6), oligomers (fractions 7–18), and aggregates (fraction 19), as listed in Table 2 (in % of total), are depicted here as histograms. Mean values and standard deviations are shown. P-values are symbolized by stars (*, ≤ 0.05; **, ≤ 0.01; ***, ≤ 0.001). The p-values indicate the significance of the differences between oligomer values of mutants and WT. Unmarked columns indicate values that are not significantly different from WT.
(TIF)

S3 Fig Distribution of GFP-tagged WT and mutant stomatin between DRMs and non-DRMs. The relative amounts of DRM-associated (fractions 1–3) and Triton X-100-soluble stomatin (fractions 4–9), as listed in Table 3 (in % of total), are depicted here as histograms. Mean values and standard deviations are shown. P-values are symbolized by stars (*, ≤ 0.05; **, ≤ 0.01; ***, ≤ 0.001). The p-values indicate the significance of the differences between values of mutants and WT. Unmarked columns indicate values that are not significantly different from WT.
(TIF)

S4 Fig Schematic structural models of mutant stomatin. Illustration of the structural consequences of deletions and point mutations. The color code and marks apply as in Fig 1. The extracellular part of the glycoprotein Pro47Ser is shown with symbolic carbohydrate chains.
(TIF)

S1 Table Mutagenic primer sequences for PCR. (PDF)

S2 Table Subcellular localization of stable stomatin mutants in A431 human carcinoma cells. (DOCX)
