# Supplementary figures and images for: Structure-function analysis of human stomatin: A mutation study
Source: PLoS One. 2017 Jun 2;12(6):e0178646. doi: 10.1371/journal.pone.0178646 (PMC5456319; doi:10.1371/journal.pone.0178646)

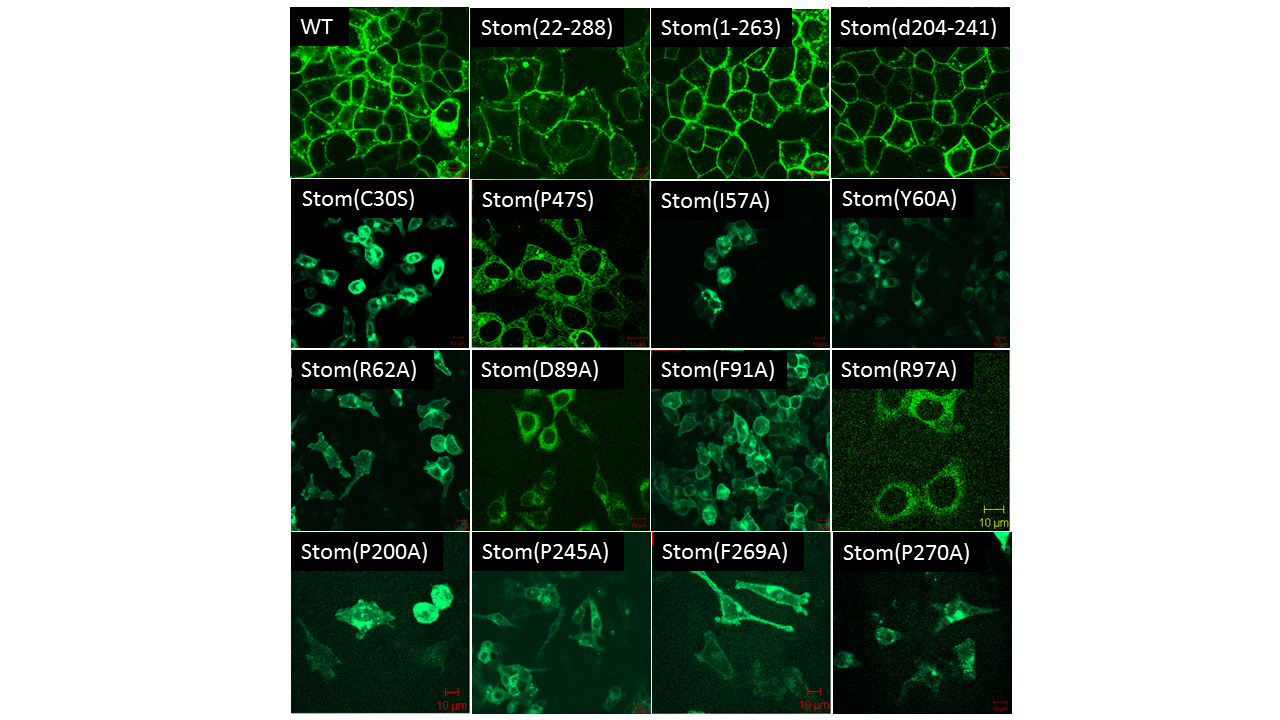

Supplement: S1 Fig — Confocal laser scanning microscopy (CLSM) of A431 cells stably expressing GFP-tagged wildtype (WT) stomatin shows the normal, dual localization to the plasma membrane (PM) and late endosomal/lysosomal compartment. Deletion of the N-terminus, C-terminus, or coiled-coil domain did not alter this localization and distribution. The point mutants Cys30Ser, Arg62Ala, Phe91Ala, and Phe269Ala, also showed the normal localization and distribution. Lack of PM staining or largely reduced staining was observed for Pro47Ser, Cys87Ser, Asp89Ala, Arg97Ala, Lys198Ala, and Pro270Ala, while weak staining of PM and preferential staining of cytoplasmic vesicles was visible in cells expressing Ile57Ala, Tyr60Ala, Pro200Ala, and Pro245Ala. (TIF) [file pone.0178646.s001.tif]

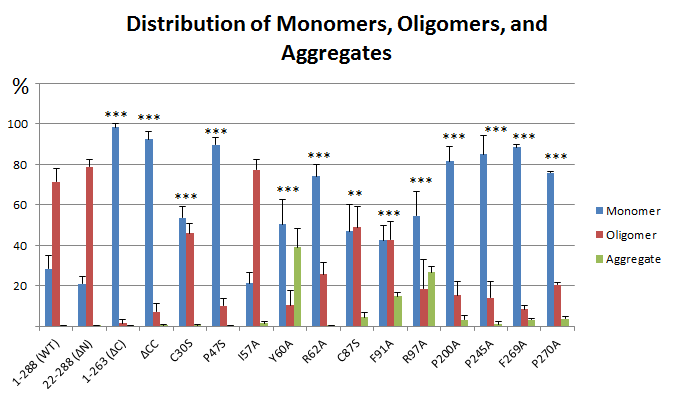

Supplement: S2 Fig — The relative amounts of mono-/dimers (fractions 1–6), oligomers (fractions 7–18), and aggregates (fraction 19), as listed in Table 2 (in % of total), are depicted here as histograms. Mean values and standard deviations are shown. P-values are symbolized by stars (*, ≤ 0.05; **, ≤ 0.01; ***, ≤ 0.001). The p-values indicate the significance of the differences between oligomer values of mutants and WT. Unmarked columns indicate values that are not significantly different from WT. (TIF) [file pone.0178646.s002.tif]

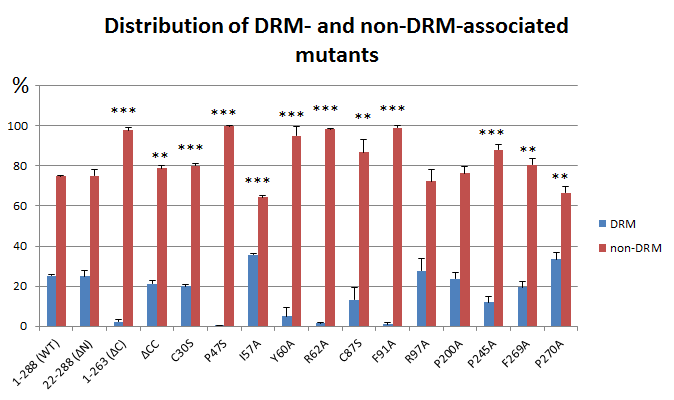

Supplement: S3 Fig — The relative amounts of DRM-associated (fractions 1–3) and Triton X-100-soluble stomatin (fractions 4–9), as listed in Table 3 (in % of total), are depicted here as histograms. Mean values and standard deviations are shown. P-values are symbolized by stars (*, ≤ 0.05; **, ≤ 0.01; ***, ≤ 0.001). The p-values indicate the significance of the differences between values of mutants and WT. Unmarked columns indicate values that are not significantly different from WT. (TIF) [file pone.0178646.s003.tif]

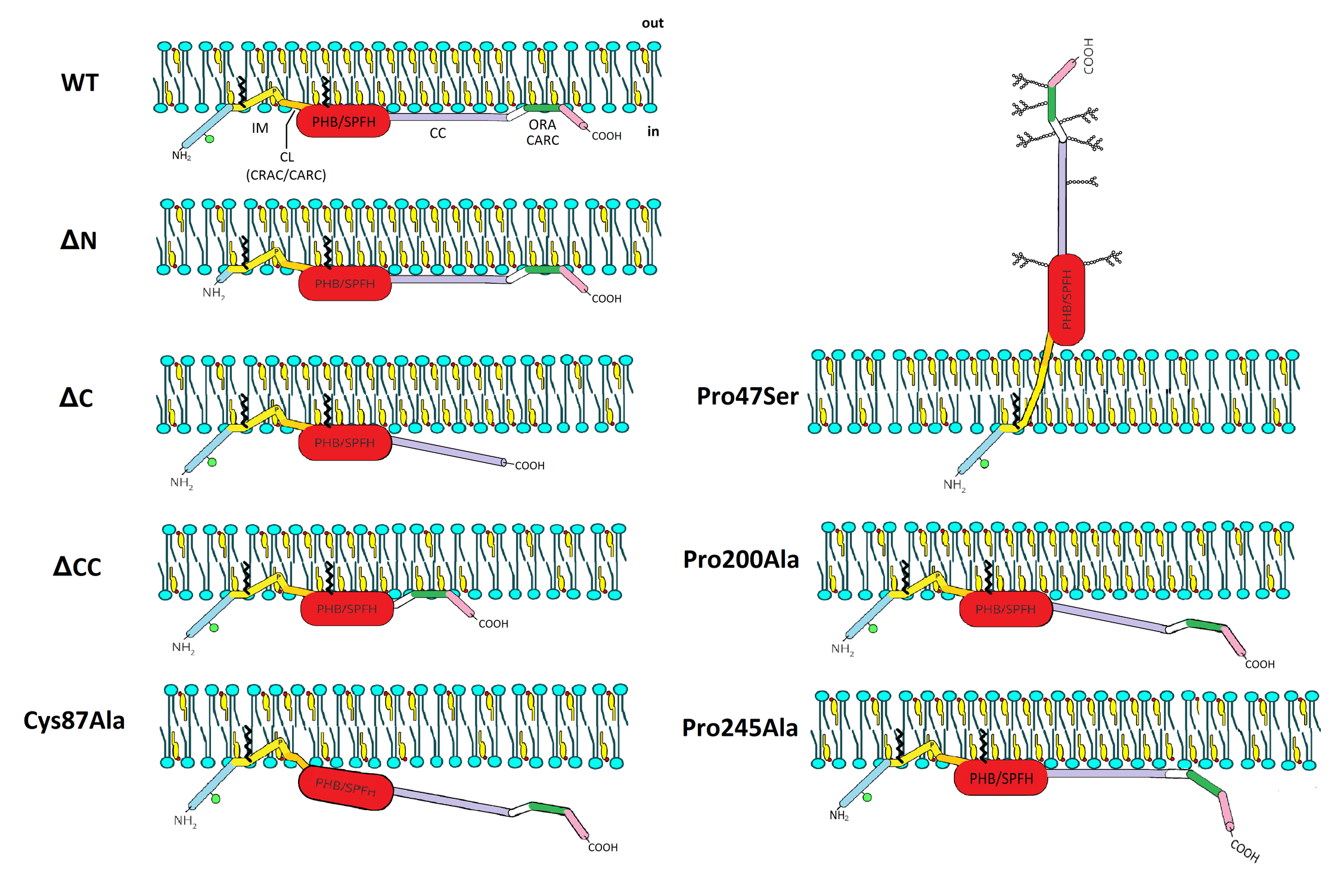

Supplement: S4 Fig — Illustration of the structural consequences of deletions and point mutations. The color code and marks apply as in Fig 1. The extracellular part of the glycoprotein Pro47Ser is shown with symbolic carbohydrate chains. (TIF) [file pone.0178646.s004.tif]
