# Supplementary material for: Structure-function analysis of human stomatin: A mutation study
Source: PLoS One. 2017 Jun 2;12(6):e0178646. doi: 10.1371/journal.pone.0178646 (PMC5456319; doi:10.1371/journal.pone.0178646)
Supplement: S1 Table — (PDF) [file pone.0178646.s005.pdf]

S1 Table. Mutagenic primer sequences for PCR.

Mutation	Clone	Mutagenic primer sequence (5'-3')
**ΔCC**	Stom(Δ204-241)GFP	F: GTGAAACTACCTGTGCAGCTCACTGAATCTCCTGCAGC<br>R: GCTGCAGGAGATTCAGTGAGCTGCACAGGTAGTTTCAC
**C30S**	Stom(C30S)GFP	F: CCTTGGACCTAGCGGATGGATTTTGGTG<br>R: CACCAAAATCCATCCGCTAGGTCCAAGG
**P47A**	Stom(P47A)GFP	F: CCGTTATAACTTTCGCAATCTCAATATGG<br>R: CCATATTGAGATTGCGAAAGTTATAACGG
**P47S**	Stom(P47S)GFP	F: CCGTTATAACTTTCTCAATCTCAATATGG<br>R: CCATATTGAGATTGAGAAAGTTATAACGG
**I57A**	Stom(I57A)GFP	F: GTGCATAAAGATTGCAAAAGAGTATG<br>R: CATACTCTTTTGCAATCTTTATGCAC
**Y60A**	Stom(Y60A)GFP	F: GATTATAAAAGAGGCTGAAAGAGCC<br>R: GGCTCTTTCAGCCTCTTTTATAATC
**Y60A+R62A**	Stom(Y60A+R62A)GFP	F: GATTATAAAAGAGGCTGAAGCAGCCATCATC<br>R: GATGATGGCTGCTTCAGCCTCTTTTATAATC
**R62A**	Stom(R62A)GFP	F: GAGTATGAAGCAGCCATCATCTTTAG<br>R: CTAAAGATGATGGCTGCTTCATACTC
**C87S**	Stom(C87S)GFP	F: ATTCTGCCAAGCACTGACAGCTTCATC<br>R: GATGAAGCTGTCAGTGCTTGGCAGAAT
**D89A**	Stom(D89A)GFP	F: CTGCCATGCACTGCAAGCTTCATCAAAG<br>R: CTTTGATGAAGCTTGCAGTGCATGGCAG
**F91A**	Stom(F91A)GFP	F: CACTGACAGCGCCATCAAAGTGG<br>R: CCACTTTGATGGCGCTGTCAGTG
**R97A**	Stom(R97A)GFP	F: GTGGACATGGCAACTATTTCATTTG<br>R: CAAATGAAATAGTTGCCATGTCCAC
**K198A**	Stom(K198A)GFP	F: GGAAATTAAGGATGTGGCACTACCTGTGC<br>R: GCACAGGTAGTGCCACATCCTTAATTTCC
**P200A**	Stom(P200A)GFP	F: GATGTGAAACTAGCTGTGCAGCTCC<br>R: GGAGCTGCACAGCTAGTTTCACATC
**P245A**	Stom(P245A)GFP	F: CACTGAATCTGCTGCAGCCCTTC<br>R: GAAGGGCTGCAGCAGATTCAGTG

**F269A**	Stom(F269A)GFP	F: CTCAACAATTGTCGCCCCTCTGCCCATAGA<br>R: TCTATGGGCAGAGGGGCGACAATTGTTGAG
**P270A**	Stom(P270A)GFP	F: TGTCTTCGCTCTGCCCATAGATATGC<br>R: GCATATCTATGGGCAGAGCGAAGACA
