# Supplementary material for: Structure-function analysis of human stomatin: A mutation study
Source: PLoS One. 2017 Jun 2;12(6):e0178646. doi: 10.1371/journal.pone.0178646 (PMC5456319; doi:10.1371/journal.pone.0178646)
Supplement: S2 Table — (DOCX) [file pone.0178646.s006.docx]

**S2 Table. Subcellular localization of stable stomatin mutants in A431 human carcinoma cells.**

| **Mutation** | **Construct** | **Affected domain** | **Localization** |
| --- | --- | --- | --- |
| **WT** | Stom(1-288)GFP | - | PM, CV |
| **ΔN** | Stom(22-288)GFP | N-terminal | PM, CV |
| **ΔC** | Stom(1-263)GFP | C-terminal | PM, CV |
| **ΔCC** | Stom(d204-241)GFP | Coiled-coil | PM, CV |
| **Cys30Ser** | Stom(C30S)GFP | IM | PM, CV |
| **Pro47Ser** | Stom(P47S)GFP | IM | CR |
| **Ile57Ala** | Stom(I57A)GFP | CRAC/CARC-like | (PM), CV |
| **Tyr60Ala** | Stom(Y60A)GFP | CRAC/CARC-like | (PM), CV |
| **Arg62Ala** | Stom(R62A)GFP | CRAC/CARC-like | PM, CV |
| **Cys87Ser** | Stom(C87S)GFP | PHB/SPFH | CV |
| **Asp89Ala** | Stom(D89A)GFP | PHB/SPFH | CV |
| **Phe91Ala** | Stom(F91A)GFP | PHB/SPFH | PM, CV |
| **Arg97Ala** | Stom(R97A)GFP | PHB/SPFH | CV |
| **Lys198Ala** | Stom(K198A)GFP | PHB/SPFH | CV |
| **Pro200Ala** | Stom(P200A)GFP | Coiled-coil | (PM), CV |
| **Pro245Ala** | Stom(P245A)GFP | Coiled-coil | (PM), CV |
| **Phe269Ala** | Stom(F269A)GFP | ORA/CARC | PM, CV |
| **Pro270Ala** | Stom(P270A)GFP | ORA/CARC | CV |

WT, wildtype; ΔN, N-terminal deletion; ΔC, C-terminal deletion; ΔCC, coiled-coil deletion; IM, intramembrane domain; CRAC/CARC, cholesterol recognition/interaction amino acid consensus sequence (direct and/or reverse motif); PHB, prohibitin homology domain; SPFH, stomatin-prohibitin- flotillin-HflK/C domain; ORA, oligomerization and lipid raft association domain; PM, plasma membrane; (PM), low expression at PM; CV, cytoplasmic vesicles; CR, cytoplasmic reticulum.
